# Artificial intelligence to predict needs for urgent revascularization from 12-leads electrocardiography in emergency patients

**DOI:** 10.1371/journal.pone.0210103

**Published:** 2019-01-09

**Authors:** Shinichi Goto, Mai Kimura, Yoshinori Katsumata, Shinya Goto, Takashi Kamatani, Genki Ichihara, Seien Ko, Junichi Sasaki, Keiichi Fukuda, Motoaki Sano

**Affiliations:** 1 Department of Cardiology, Keio University School of Medicine, Tokyo, Japan; 2 Department of Medicine (Cardiology), Tokai University School of Medicine, Isehara, Japan; 3 Division of Pulmonary Medicine, Department of Internal Medicine, Keio University School of Medicine, Tokyo, Japan; 4 Department of Medical Science Mathematics, Tokyo Medical and Dental University, Tokyo, Japan; 5 Department of Emergency and Critical Care Medicine, Keio University School of Medicine, Tokyo, Japan; Niigata Daigaku, JAPAN

## Abstract

**Background:**

Patient with acute coronary syndrome benefits from early revascularization. However, methods for the selection of patients who require urgent revascularization from a variety of patients visiting the emergency room with chest symptoms is not fully established. Electrocardiogram is an easy and rapid procedure, but may contain crucial information not recognized even by well-trained physicians.

**Objective:**

To make a prediction model for the needs for urgent revascularization from 12-lead electrocardiogram recorded in the emergency room.

**Method:**

We developed an artificial intelligence model enabling the detection of hidden information from a 12-lead electrocardiogram recorded in the emergency room. Electrocardiograms obtained from consecutive patients visiting the emergency room at Keio University Hospital from January 2012 to April 2018 with chest discomfort was collected. These data were splitted into validation and derivation dataset with no duplication in each dataset. The artificial intelligence model was constructed to select patients who require urgent revascularization within 48 hours. The model was trained with the derivation dataset and tested using the validation dataset.

**Results:**

Of the consecutive 39,619 patients visiting the emergency room with chest discomfort, 362 underwent urgent revascularization. Of them, 249 were included in the derivation dataset and the remaining 113 were included in validation dataset. For the control, 300 were randomly selected as derivation dataset and another 130 patients were randomly selected for validation dataset from the 39,317 who did not undergo urgent revascularization. On validation, our artificial intelligence model had predictive value of the c-statistics 0.88 (95% CI 0.84–0.93) for detecting patients who required urgent revascularization.

**Conclusions:**

Our artificial intelligence model provides information to select patients who need urgent revascularization from only 12-leads electrocardiogram in those visiting the emergency room with chest discomfort.

## Introduction

Patient with acute coronary syndrome (ACS) benefits from early revascularization with percutaneous coronary intervention or coronary artery bypass grafting [1, 2]. At the emergency room (ER), multiple information including symptoms, bio-markers, and 12-leads electrocardiogram (ECG) are used to select the patients requiring urgent revascularization. Previous studies demonstrated that from 1% to 6% of ACS patients present with “normal ECG” [3–5]. Current high-performance computer technology and artificial intelligent (AI) may be able to pick up information that reflects whether the patients require urgent revascularization or not.

Here we report an AI model trained by consecutive ECG taken at the ER in a single medical center to select patients who require urgent revascularization.

## Methods

### Outcome definitions

The outcome of the current analysis was the need for urgent revascularization within 48 hours from the initial visit to the hospital.

### Patient selection

**Fig 1:**
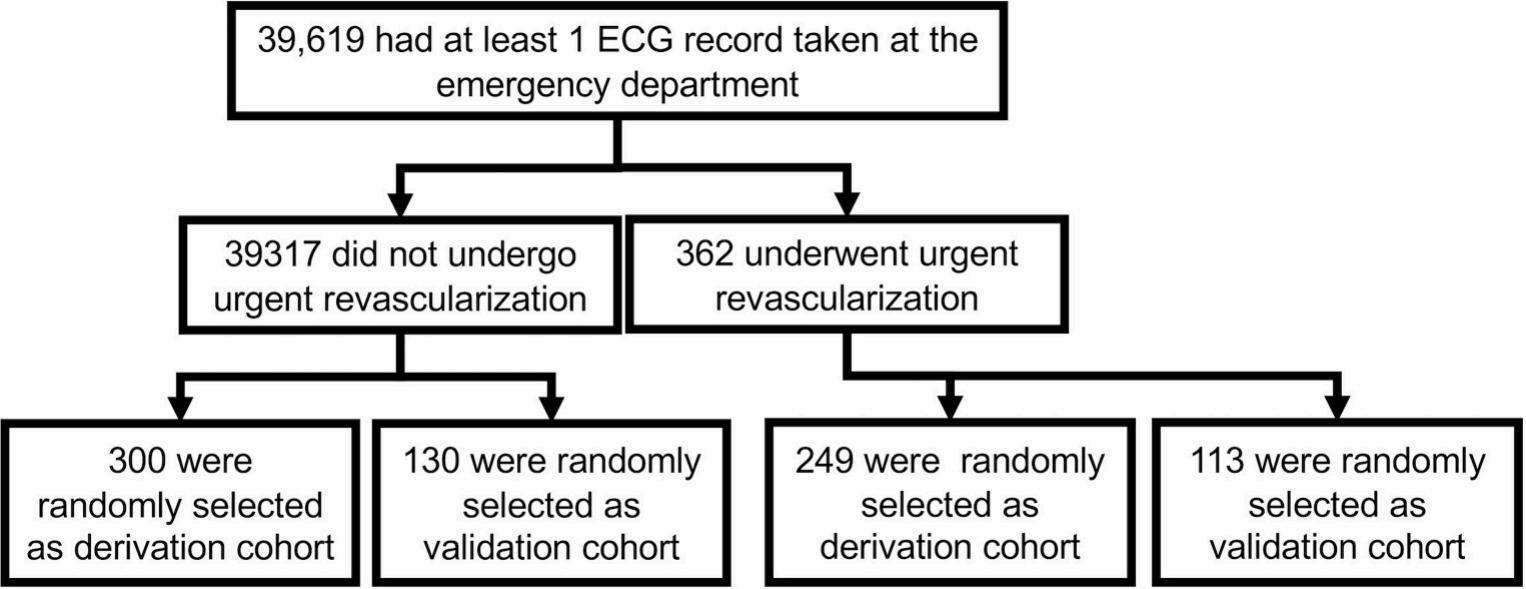
Selection of study population. ECG: electrocardiogram.

The patient selection process is shown in Fig 1. Consecutive patients who presented to the ER of Keio University hospital and who had at least one ECG recorded on the day of visit within January 2012 to April 2018 were included in the analysis. ECG from patients who underwent urgent revascularization and those who were randomly selected from patients who have not underwent urgent revascularization were included in the analysis. These patients were again randomly splitted into the derivation cohort and the validation cohort.

The study protocol was approved by institutional review board of Keio University Hospital (20170204). Our study complies with the necessary regulatory requirement.

### Al model

**Fig 2:**
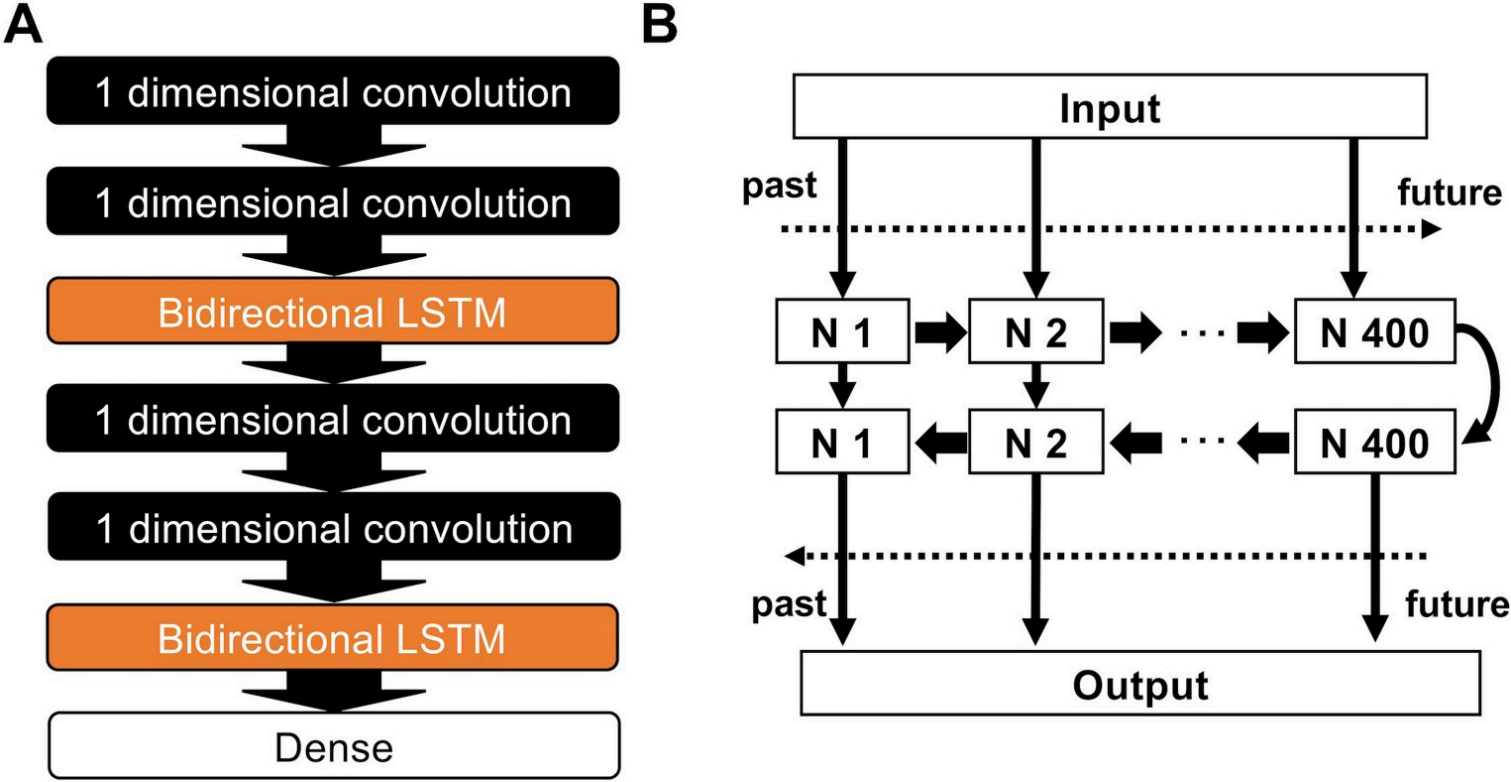
Structure of the neural network in our AI model. Schematic illustration of the neural network model (A). Schematic illustration of bidirectional LSTM(B). Note that two layers of LSTM which have opposite directions of information transfer with the neurons next to each other are stacked up. LSTM: long short-term memory. N: neuron.

The structure of neural networks in the AI model is shown in Fig 2A. Since the ECG is a time-series data of voltage, the AI model was constructed by stacking up multiple layers of special neurons that can deal with time-dependent data, namely one-dimensional convolution layer and bidirectional long short-term memory (LSTM) layer. The LSTM layer has rectified data transfer to the neuron next to each other[6]. By doing so, this layer can learn time-dependent data in its order. The bidirectional LSTM layer stacks up two of these LSTM layers, which has the opposite rectified data transfer (Fig 2B). This allows the LSTM to learn data in two directions (past to the future and future to the past).

**Fig 3:**
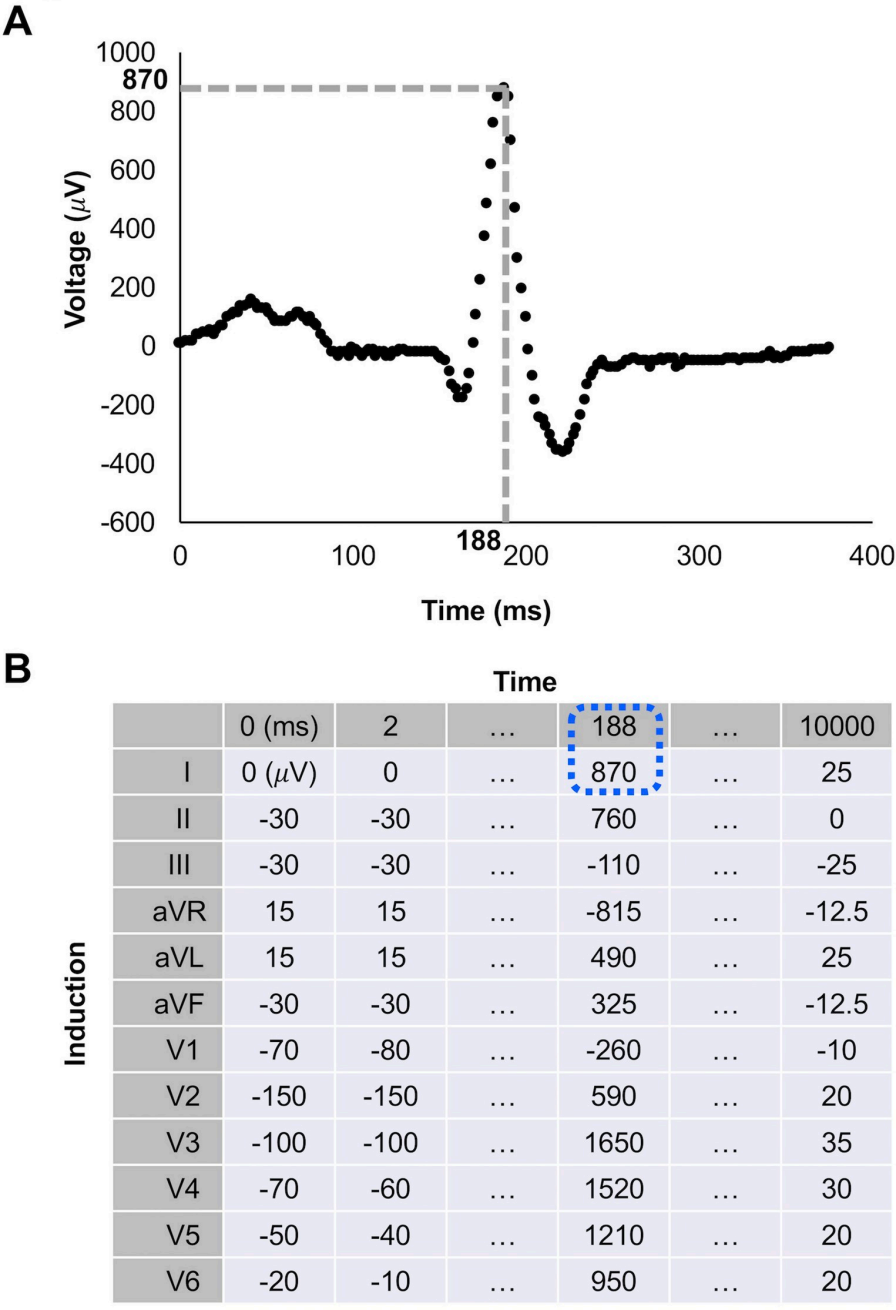
Conversion of ECG data to 2D matrix. A representative plot of a single beat at induction I picked up from a 12-lead ECG recording (A). The recorded data consists of voltage plotted against time. A representative 2-dimention matrix converted from the 12-lead ECG recording (B). The matrix has 2 axis of induction axis and time axis. The value at the point indicated with dotted grey line in A converted to an element in the matrix is highlighted with dotted blue line in B. Voltage for each induction was recorded in each 2 ms. ECG: electrocardiogram.

The model was trained using the 12-lead ECG data recorded for 10 seconds at rest, converted to a 2-dimensional matrix with the time axis and the induction axis containing the recorded voltage (Fig 3). Training was done using only the data of patient from derivation cohort (n = 249 for patients who underwent urgent revascularization and 300 for patients who did not require urgent revascularization). The input of the model was the ECG labeled by either urgent revascularization was performed or not in each individual patient.

### Validation of the Al model

The derived model was validated by comparing the predicted outcome with the actual clinical course for each individual patient in validation cohort. Receiver operating characteristic (ROC) curve was compiled to evaluate the predictive value of the model. The threshold for the best accuracy of the model was calculated. Sensitivity and specificity were calculated at that threshold. The probability of the necessity of urgent revascularization was calculated for each quartile of the output of our model.

### Statistical analysis

The neural network was constructed and trained using the Keras framework [7] using TensorFlow [8] as backend. The neural network was trained using the back-propagation supervised training algorithm. The loss function of binary cross entropy was minimized using the RMSprop optimizer.

The c-statistics, best accuracy, threshold, sensitivity and specificity of the model and its 95% confidence interval (CI) were calculated using the bootstrap procedure with 2000 bootstrap rounds using the pROC package of R[9].

The statistical analysis of probability of urgent revascularization with each quartile of model output was done using R version 3.5.1. Fisher’s exact test was used to calculate the p value. P value <0.05 were considered as statistically significant.

## Results

### Patients

During January 2012 to April 2018, 39,619 patients visited the ER of Keio University hospital and had at least one 12-lead ECG recording at the day of visit. Of them, 362 underwent urgent revascularization within 48 hours and 39,317 did not. From these patients, 249 patients who underwent urgent revascularization and 300 patients who did not were randomly selected and were included in the derivation cohort. The remaining 113 patients who underwent urgent revascularization and another randomly selected 130 patients without urgent revascularization (no duplication with the 300 patients included in derivation cohort) were included in the validation cohort.

### Predictive value of the AI model

**Fig 4:**
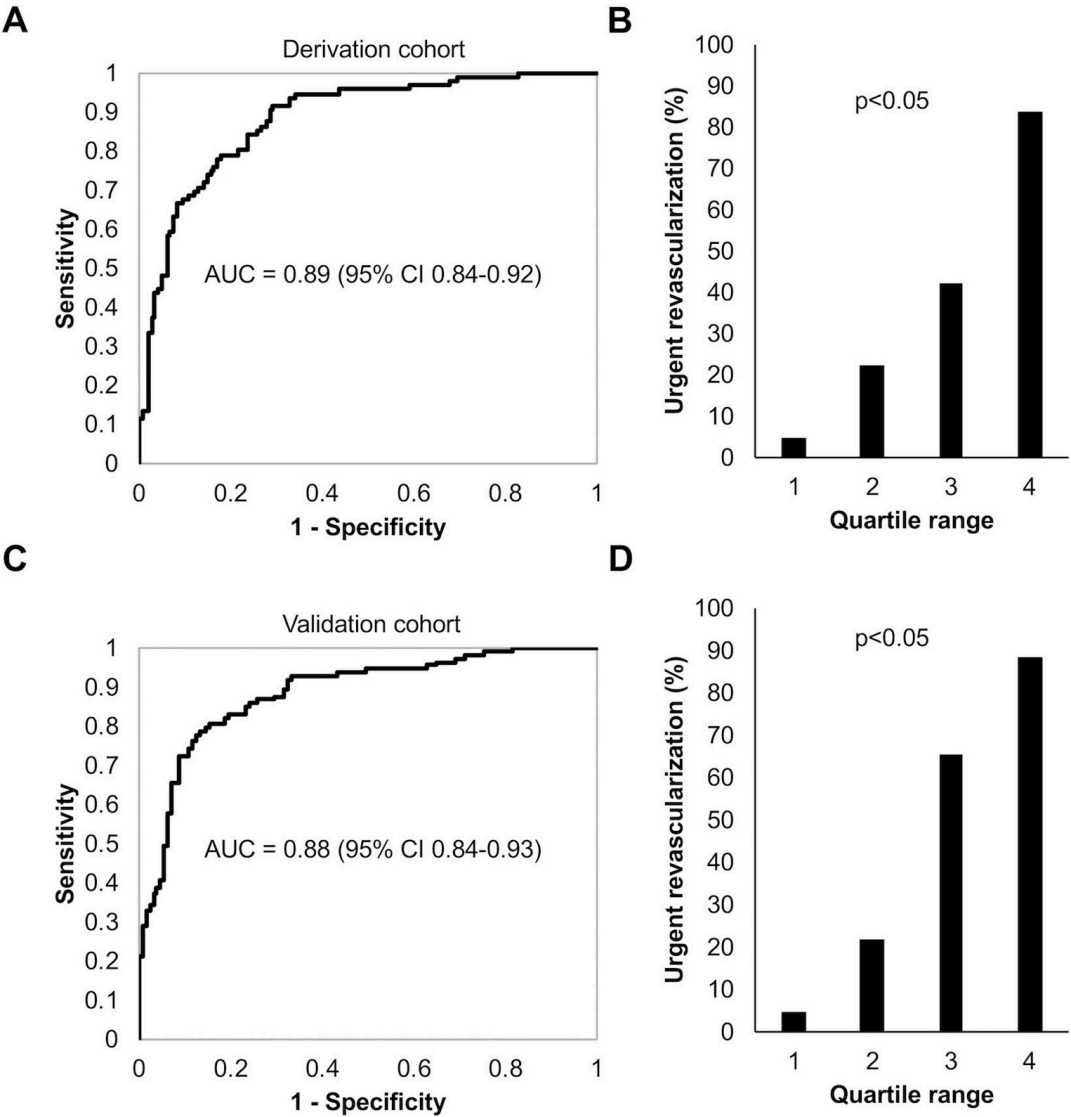
Diagnostic value of the AI model. ROC curve (A) and probability of receiving urgent revascularization for patients stratified to each quartile range of the model output using the derivation cohort(B). The results from same analysis using validation cohort are shown in panel C and D. The p values were calculated using Fisher’s exact test. ROC: receiver operating characteristic. AUC: area under curve. CI: confidence interval.

The analysis of ROC curve of our model for derivation cohort (Fig 4A) revealed that our model had a predictive value of c-statistics of 0.89 (95% CI 0.84–0.92) for selecting patients who required urgent revascularization. The probability of patients who underwent urgent revascularization for each quartile of the model output (Fig 4B) showed significantly higher rates with high quartile range values.

On validation with, our AI model showed similar c-statistics of 0.88 (95% CI 0.84–0.93) and the analysis of probability of undergoing urgent revascularization for each quartile range was similar with high rate of urgent revascularization with higher quartiles (Fig 4C and 4D). The ranges of model outputs for each quartile for validation cohort are summarized in Table 1. The best accuracy of classification was 0.83 (95% CI 0.79–0.88) with the sensitivity and specificity of 0.79 (95% CI 0.71–0.86) and 0.87 (95% CI 0.81–0.92), respectively (Table 2).

**Table 1:** The value of model output for each quartile ranges.

Quartile range	Model output
1st	0.00–0.36
2nd	0.36–0.44
3rd	0.44–0.53
4th	0.53–1.00

**Table 2:** Results with threshold giving the best accuracy for validation cohort.

	Value (95% CI)
Accuracy	0.83 (0.79–0.88)
Sensitivity	0.79 (0.71–0.86)
Specificity	0.87 (0.81–0.92)

## Discussion

Our results suggest that the AI model constructed with neural networks including 1-dimensional convolution and bidirectional LSTM pick up information from 12-leads ECG to predict whether the patients presented with chest discomfort to the ER needs urgent revascularization or not. The most unique part of our analysis is the development of prediction model with 12-leads ECG alone, without using other known parameters such as serological bio-markers. Our results strongly suggest the presence of specific ECG characteristics not recognized by physician but can be detected by the AI.

There are multiple applications of AI using neural networks in the field of cardiology[10–14]. However, only limited reports are available so far for its application to 12-lead ECGs[15–17]. One article by Myers et al[16]. have shown that machine learning with recurrent neural network combined with logistic regression of conventional risk factors can predict all cause death in (non-ST-elevation ACS) NSTE-ACS patients. Successful prediction with their model should depend on the use of recurrent neural network (RNN). The weakness of their model is that the application is only limited in patients diagnosed as NSTE-ACS. The mortality could be predictable by their model, but the likelihood for urgent revascularization is still to be elucidated.

At the ER, it is particularly important to select patients who need urgent revascularization when they presented with the symptom of chest discomfort. Various biomarkers in addition to patients’ symptom are proven to be effective to select patients with acute coronary syndrome requiring urgent revascularization. We hypothesized that 12-lead ECG at emergency room contain information necessary to discriminate patients who need urgent revascularization from those who don’t. To test this hypothesis, the AI model we have developed was trained only by ECG data and clinical consequence of urgent revascularization yes/no. Indeed, the AI were able to predict the clinical course whether the patients need urgent revascularization or not with c-statistic of 0.89 (95% CI 0.84–0.92). This result strongly suggest that the AI detected information not detected by human eye or previously available pattern recognition that related to the presence of acute myocardial ischemia.

Our AI model comprised of a neural network model including multiple 1-dimentional convolution layers and LSTM layers. The LSTM layers are strong to deal with time-dependent data. On the other hand, LSTM requires massive computational resources. The 1-dimentional convolution can compress the time dependent data into shorter matrix, thus it enables the LSTM to save computational resource. The combination of convolution network and LSTM enabled compression of large data with 1-dimentional convolution layers and then let the LSTM to learn the complex pattern.

To date, there are several models to predict the need of urgent revascularization in chest pain patients[18]. Of them, the diagnostic accuracy of TIMI risk score in chest pain patients is widely used[19]. The sensitivity and specificity of TIMI risk score were reported as 97% and 25%, respectively for acute myocardial infarction, coronary revascularization or death from any cause respectively. Our model using only 12-lead ECG had a similar diagnostic value with a specificity of 29% when the sensitivity is 97% according to the ROC curve. Previous reports showed that interpretation of ECG by a cardiologist showed sensitivity and specificity of 51.5% and 66.1% respectively[20] while our model can achieve 94% specificity when the sensitivity is 51.5%. It is of note that neither biomarker or baseline characteristic were not included in our model. It is of particular interest, that only one time of 12-lead ECG at arrival contain important information for clinical decision making.

Several limitations of the current analysis should be noted. Firstly, the current analysis was performed in a single center in Japan. Our hospital is a University hospital located in the city center of an urban area of Japan. The selection of patients who need urgent revascularization in the hospital may be biased. Further external validation analysis using external datasets are necessary to establish the validity of our AI model in the world. Second, our AI was trained only with ECG data, but did not include others such as age, sex, biomarkers, or concomitant drugs. Though we have analyzed consecutive patient data, one might argue that there are potential and unrecognized confounder for selecting patients need for urgent coronary revascularization. Third, our results suggest the presence of crucial information within ECG to predict the clinical course of patient, but not providing the information which segment of ECG contain that kind of information. The information might be present in atrial contraction/relaxation part, ventricular contraction/relaxation part, or other part. Finally, our model only included the ECG data. Including other patient data could have further improved the diagnostic value of our model.

## Conclusion

Our AI model suggest the presence of crucial information in 12-lead ECG, taken from patients presenting with chest discomfort to the ER, to predict the future clinical course.

## Supporting information

S1 File Explanation for the use of prediction model. Explanation of how to use the python source code and the weights of AI model.
(DOCX)

S2 File Python source code of the AI model. A python source code of the AI model for calculating the need of urgent revascularization from ECG data.
(PY) Click here for additional data file.

S3 File Model weights. The weights of the model to calculate the need of urgent revascularization.
(HDF5)

S4 File Raw data for ROC analysis in derivation cohort. The sensitivities and specificities of the model for derivation cohort.
(XLSX)

S5 File Raw data for ROC analysis in validation cohort. The sensitivities and specificities of the model for validation cohort.
(XLSX)

S6 File Raw data for quartile analysis in derivation cohort. The raw calculation sheet for the quartile analysis in derivation cohort.
(XLSX)

S7 File Raw data for quartile analysis in validation cohort. The raw calculation sheet for the quartile analysis in validation cohort.
(XLSX)
