# Supplementary material for: Artificial intelligence to predict needs for urgent revascularization from 12-leads electrocardiography in emergency patients
Source: PLoS One. 2019 Jan 9;14(1):e0210103. doi: 10.1371/journal.pone.0210103 (PMC6326503; doi:10.1371/journal.pone.0210103)
Supplement: S1 File — Explanation of how to use the python source code and the weights of AI model. (DOCX) [file pone.0210103.s001.docx]

# Python source code to run the AI model

The python source code of the AI model is provided as S2 File. The weights of the models to predict the outcomes are provided as S3 File. The input data should contain the voltage in the unit of 125 𝜇V. The length of recording should be 10 seconds and the voltage should be recorded each 2 ms. The data should be stored in the numpy matrix format: first axis is for patient, second for the lead of I, II, III, aVR, aVL, aVF, V1, V2, V3, V4, V5 and V6 in this order and the third axis is for time.
